# Supplementary material for: Differing isoforms of the cobalamin binding photoreceptor AerR oppositely regulate photosystem expression
Source: eLife. 2018 Oct 3;7:e39028. doi: 10.7554/eLife.39028 (PMC6199135; doi:10.7554/eLife.39028)
Supplement: Supplementary file 5. [file elife-39028-supp5.docx]

Supplementary File 5. List of primers in this study

| Primer name | Sequence (5’-3’) | Use |
| --- | --- | --- |
| Rc_aerR-f | TCGGGGACTCGAATGCGGGATATAAGTGTTGAACTC | aerR expression |
| pSRK-pBBR-r | GGTATCGATAAGCTTATCGAATTCCTGCAGCCCGG | aerR expression |
| Rc_aerRup-f | CAGGAATTCGATATCGGAACTGCAGCGGGGCCAGG | aerR expression |
| Rc_aerRup-r | CATTCGAGTCCCCGAAACCCAACG | aerR expression |
| Rc_aerR-insA-f | TCGGGGACTCGAATGACGGGATATAAGTGTTGAACTC | M1a-AerR expression |
| Rc_aerR_MNG-f | TCGGGGACTCGAATGAACGGAGTGGCGGAACTGCAG | M35-AerR expression |
| Rc_aerR_MVE-f | TCGGGGACTCGAATGGTCGAACTGGCAACGGGACC | M49-AerR expression |
| Rc_aerR_MDL-f | TCGGGGACTCGAATGGACCTGCTGTTCGACGAATTC | M61-AerR expression |
| Rc_aerR_V38A-f | AACGGAGCGGCGGAACTGCAGACCGTGC | V38A substitution |
| Rc_aerR_V38A-r | TTCCGCCGCTCCGTTCATGCGCCGCGCG | V38A substitution |
| Rc_aerR_E40A-f | TGGCGGCGCTGCAGACCGTGCTTGTGAAC | E40A substitution |
| Rc_aerR_E40A-r | TCTGCAGCGCCGCCACTCCGTTCATGCGC | E40A substitution |
| Rc_aerR_L41A-f | GCGGAAGCGCAGACCGTGCTTGTGAACAG | L41A substitution |
| Rc_aerR_L41A-r | GGTCTGCGCTTCCGCCACTCCGTTCATGC | L41A substitution |
| Rc_aerR_Q42A-f | GAACTGGCGACCGTGCTTGTGAACAGGATG | Q42A substitution |
| Rc_aerR_Q42A-r | CACGGTCGCCAGTTCCGCCACTCCGTTCATG | Q42A substitution |
| Rc_aerR_T43A-f | ACTGCAGGCCGTGCTTGTGAACAGGATG | T43A substitution |
| Rc_aerR_T43A-r | AGCACGGCCTGCAGTTCCGCCACTCCGTTC | T43A substitution |
| Rc_aerR_V44A-f | CAGACCGCGCTTGTGAACAGGATGGTCG | V44A substitution |
| Rc_aerR_V44A-r | CACAAGCGCGGTCTGCAGTTCCGCCACTC | V44A substitution |
| Rc_aerR_L45A-f | ACCGTGGCGGTGAACAGGATGGTCGAACTG | L45A substitution |
| Rc_aerR_L45A-r | GTTCACCGCCACGGTCTGCAGTTCCGCCAC | L45A substitution |
| Rc_aerR_M35ai ns-f | GCGCATGAAACGGAGTGGCGGAACTGCAG | a insertion after M35 codon |
| Rc_aerR_M35ai ns-r | CTCCGTTTCATGCGCCGCGCGACAAGCC | a insertion after M35 codon |
| Rc_aerR_V38ain s-f | CGGAGTGAGCGGAACTGCAGACCGTGCTTG | a insertion after V38 codon |

| Primer name | Sequence (5’-3’) | Use |
| --- | --- | --- |
| Rc_aerR_V38ain s-r | GTTCCGCTCACTCCGTTCATGCGCCGCG | a insertion after V38 codon |
| Rc_aerR_L41ain s-f | GGAACTGACAGACCGTGCTTGTGAACAG | a insertion after L41 codon |
| Rc_aerR_L41ain s-r | GGTCTGTCAGTTCCGCCACTCCGTTCATG | a insertion after L41 codon |
| Rc_aerR_T43ain s-f | GCAGACCAGTGCTTGTGAACAGGATGGTC | a insertion after T43 codon |
| Rc_aerR_T43ain s-r | CAAGCACTGGTCTGCAGTTCCGCCACTC | a insertion after T43 codon |
| Rc_aerR_V44ain s-f | GACCGTGACTTGTGAACAGGATGGTCGAAC | a insertion after V44 codon |
| Rc_aerR_V44ain s-r | TCACAAGTCACGGTCTGCAGTTCCGCCAC | a insertion after V44 codon |
| Flag-bchE-f | GACTACAAAGACCATGACGGTG | reporter assay |
| Rc_aerR_A39- FbchE-r | ATGGTCTTTGTAGTCCGCCACTCCGTTCATGCGCC | reporter assay |
| Rc_aerR_E40- FbchE-r | ATGGTCTTTGTAGTCTTCCGCCACTCCGTTCATGC | reporter assay |
| Rc_aerR_L41- FbchE-r | ATGGTCTTTGTAGTCCAGTTCCGCCACTCCGTTCATG | reporter assay |
| M13+pBBR- MSC2-r | GGTATCGATAAGCTTGTAAAACGACGGCCAGTGAATTG | reporter assay |
| pBBR1MCS-2-f | AAGCTTATCGATACCGTCGACC | reporter assay |
| Rc_aerR_L41A2- f | GCGGAAGCCCAGACCGTGCTTGTGAACAG | L41A substitution |
| Rc_aerR_L41A2- r | GGTCTGGGCTTCCGCCACTCCGTTCATGC | L41A substitution |
| Rc_aerR_L41L-f | GCGGAATTGCAGACCGTGCTTGTGAACAG | L41L substitution |
| Rc_aerR_L41L-r | GGTCTGCAATTCCGCCACTCCGTTCATGC | L41L substitution |
| Rc_aerR_L41I-f | GCGGAAATCCAGACCGTGCTTGTGAACAG | L41I substitution |
| Rc_aerR_L41I-r | GGTCTGGATTTCCGCCACTCCGTTCATGC | L41I substitution |
| Rc_aerR_L41V-f | GCGGAAGTCCAGACCGTGCTTGTGAACAG | L41V substitution |
| Rc_aerR_L41V-r | GGTCTGGACTTCCGCCACTCCGTTCATGC | L41V substitution |
| Rc_aerR_L41P-f | GCGGAACCCCAGACCGTGCTTGTGAACAG | L41P substitution |
| Rc_aerR_L41P-r | GGTCTGGGGTTCCGCCACTCCGTTCATGC | L41P substitution |

| Primer name | Sequence (5’-3’) | Use |
| --- | --- | --- |
| Rc_aerR_L41F-f | GCGGAATTCCAGACCGTGCTTGTGAACAG | L41F substitution |
| Rc_aerR_L41F-r | GGTCTGGAATTCCGCCACTCCGTTCATGC | L41F substitution |
| pZJD29_SphI-f | CCTGCAGGTCGACTCTAGAG | chromosome transformation |
| pZJD29_SphI-r | CATGCAAGCTTGGCGTAATC | chromosome transformation |
| Rc_aerRup-f2 | CGCCAAGCTTGCATGGGAACTGCAGCGGGGCCAGG | chromosome transformation |
| Rc_aerR1130-r | GAGTCGACCTGCAGGTCAGGCTGGACAGGGTTTTG | chromosome transformation |
| Rc_aerR_G37+L 41-f1 | GGTGTGGCGGAACTCCAGACCGTGCTTGTGAACAG | Silent mutations on G37 and L41 codons |
| Rc_aerR_G37+L 41-r1 | GAGTTCCGCCACACCGTTCATGCGCCGCGCGACAAG | Silent mutations on G37 and L41 codons |
| Rc_aerR_G37+L 41-f2 | GGTGTGGCGGAACTTCAGACCGTGCTTGTGAACAG | Silent mutations on G37 and L41 codons |
| Rc_aerR_G37+L 41-r2 | AAGTTCCGCCACACCGTTCATGCGCCGCGCGACAAG | Silent mutations on G37 and L41 codons |
| Rc_saerR  +SUMO-f | CAGATTGGAGGTATGCTGCAGACCGTGCTTGTGAAC | SAerR overexpression |
| Rc_saerR  +SUMO-r | AAGCACGGTCTGCAGCATACCTCCAATCTGTTCGC | SAerR overexpression |
| Rc_aerR_G148E- f | CAGGCCACGCTGGAGGCGTTGATCGTGGCGATGGAAC | G148E substitution |
| Rc_aerR_G148E- r | CCACGATCAACGCCTCCAGCGTGGCCTGCTCTCCG | G148E substitution |

Highlighted sequences in red indicate substitution or insertion to original aerR sequence.
